# Anti-drug Antibodies Against a Novel Humanized Anti-CD20 Antibody Impair Its Therapeutic Effect on Primary Biliary Cholangitis in Human CD20- and FcγR-Expressing Mice

**DOI:** 10.3389/fimmu.2018.02534

**Published:** 2018-11-02

**Authors:** Yuki Moritoki, Koichi Tsuneyama, Yuka Nakamura, Kentaro Kikuchi, Akira Shiota, Yoshiyuki Ohsugi, Zhe-Xiong Lian, Weici Zhang, Guo-Xiang Yang, Shigeharu Ueki, Masahide Takeda, Ayumi Omokawa, Tomoo Saga, Akiko Saga, Daisuke Watanabe, Masahito Miura, Yoshiyuki Ueno, Patrick S. C. Leung, Atsushi Tanaka, M. Eric Gershwin, Makoto Hirokawa

**Affiliations:** ^1^Department of General Internal Medicine and Clinical Laboratory Medicine, Akita University Graduate School of Medicine, Akita, Japan; ^2^Center for Medical Education and Training, Akita University Hospital, Akita, Japan; ^3^SimTiki Simulation Center, John A. Burns School of Medicine, University of Hawaii, Honolulu, HI, United States; ^4^Department of Pathology and Laboratory Medicine, Institute of Biomedical Science, Tokushima University Graduate School of Medicine, Tokushima, Japan; ^5^Department of Fourth Internal Medicine, Teikyo University Mizonokuchi Hospital, Kawasaki, Japan; ^6^Institute of Immunology, Co., Ltd., Tokyo, Japan; ^7^Ohsugi BioPharma Consulting, Co., Ltd., Tokyo, Japan; ^8^Chronic Disease Laboratory, Institutes for Life Sciences and School of Medicine, South China University of Technology, Guangzhou, China; ^9^Division of Rheumatology, Allergy and Clinical Immunology, Genome and Biomedical Sciences Facility, University of California, Davis, Davis, CA, United States; ^10^Watanabe Internal Medicine Clinic, Noshiro, Japan; ^11^Department of Gastroenterology, Omagari Kosei Medical Center, Omagari, Japan; ^12^Department of Gastroenterology, Yamagata University Faculty of Medicine, Yamagata, Japan; ^13^Department of Medicine, Teikyo University School of Medicine, Tokyo, Japan

**Keywords:** anti-drug antibodies (ADAs), anti-mitochondrial antibodies (AMAs), B cell depletion therapy, human anti-chimeric antibodies (HACAs), humanized anti-human CD20 antibody, mouse anti-human antibodies (MAHAs), primary biliary cholangitis (PBC)

## Abstract

There is considerable interest in expanding B cell-targeted therapies in human autoimmune diseases. However, clinical trials in human primary biliary cholangitis (PBC) using a chimeric antibody against human CD20 (hCD20) have showed limited efficacy. Two potential explanations for these disappointing results are the appearance of anti-drug antibodies (ADAs) and the high frequency of patients with moderate PBC or patients who had failed ursodeoxycholic acid treatment. Here, we studied a novel humanized IgG1 antibody against hCD20 and explored its efficacy in early stage PBC using a well-defined murine model. We developed a unique murine model consisting of dnTGF-βRII mice expressing hCD20 and human Fcγ receptors (hFcγRs). Beginning at 4–6 weeks of age, equivalent to stage I/II human PBC, female mice were given weekly injections of an anti-hCD20 antibody (TKM-011) or vehicle control, and monitored for liver histology as well as a broad panel of immunological readouts. After 16 weeks' treatment, we observed a significant reduction in portal inflammation, a decrease in liver-infiltrating mononuclear cells as well as a reduction in liver CD8^+^ T cells. Importantly, direct correlations between numbers of liver non-B cells and B cells (*r* = 0.7426, *p* = 0.0006) and between numbers of liver memory CD8^+^ T cells and B cells (*r* = 0.6423, *p* = 0.0054) were apparent. Accompanying these changes was a dramatic reduction in anti-mitochondrial antibodies (AMAs), interleukin (IL)-12p40 and IL-5, and elevated levels of the anti-inflammatory chemokine CXCL1/KC. In mice that developed ADAs, clinical improvements were less pronounced. Sustained treatment with B cell-targeted therapies may broadly inhibit effector pathways in PBC, but may need to be administered early in the natural history of PBC.

## Introduction

The destruction of biliary epithelial cells (BECs) in patients with primary biliary cholangitis (PBC) is at least partially secondary to development of autoreactive CD8^+^ T cells (1–3). In addition, there is evidence that B cells and serum anti-mitochondrial antibodies (AMAs) exacerbate biliary pathology through their effects on apoptotic biliary cells as well as through B-cell regulatory mechanisms; inflammatory liver infiltrates include B-cell foci (4). Although there is no direct correlation between AMA titer and disease severity, a variety of data support a role of B cells in the immunopathology of PBC (5–10). For example, dimeric IgA-AMA complexes facilitate induction of BEC apoptosis (8) and AMAs enhance cross-presentation and generation of pyruvate dehydrogenase complex-E2 (PDC-E2)-specific cytotoxic T cell responses in the presence of PDC-E2-pulsed antigen-presenting cells (5). In combination with AMAs, unmodified PDC-E2 localized in apoptotic BECs facilitates production of proinflammatory cytokines from monocyte-derived macrophages in PBC (6, 7). These results have led us to postulate that the cellular responses involved in loss of tolerance and biliary pathology include contributions from both B and T cells and that biliary destruction is an orchestrated multi-lineage response (11).

We have previously reported the use of dominant negative TGF-β receptor II (dnTGF-βRII) mice as a model for human PBC (12). The expression of dnTGF-βRII is limited to selected cell lineages including CD4^+^, CD8^+^, and CD1d-restricted natural killer T (NKT) cells; B cell function is normal in this model (12, 13). We previously demonstrated that treatment of juvenile mice with a monoclonal anti-mouse CD20 antibody was effective in preventing PBC, but that responses were reduced when treatment was initiated in older mice (14). This may reflect a limited contribution by B cells in the pathology of advanced bile duct damage during human PBC (15).

**Figure 1:**
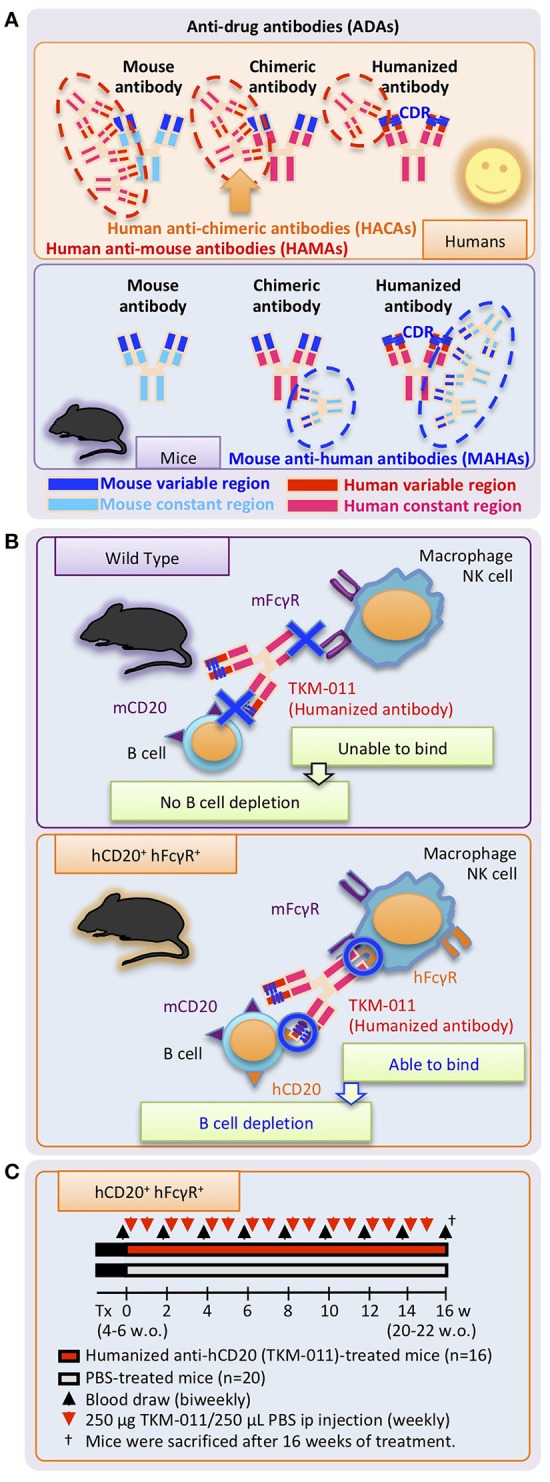
Study design. **(A)** Undesirable immune responses result in development of anti-drug antibodies (ADAs) against therapeutic monoclonal antibodies. Specific regions of chimeric antibodies (murine variable and human constant regions) and humanized antibodies (human with murine complementary-determining regions, CDRs) are immunogenic in humans and mice, and are targeted by human anti-mouse antibodies (HAMAs) including human anti-chimeric antibodies (HACAs) and mouse anti-human antibodies (MAHAs) in humans and mice, respectively. **(B)** Working mechanisms of the humanized anti-human CD20 mAb, TKM-011, in human CD20 (hCD20)-, and human FcγR (hFcγR)-expressing mice. Genetic modification enabled hCD20 and hFcγR expression under the control of mouse CD20 and mouse FcγR promoters, respectively. TKM-011 binds to hCD20 and is captured by hFcγR-expressing cells, leading to B-cell elimination mediated by antibody-dependent cell-medicated cytotoxicity (ADCC). **(C)** Mice expressing hCD20 and hFcγR were injected with 250 μg of TKM-011 or 250 μL of PBS intraperitoneally every week beginning at 4–6 weeks of age. Peripheral blood was drawn for serum collection and lymphocyte analyses biweekly prior to each treatment. Mice were sacrificed after 16 weeks of treatment, and their tissues were used for pathological evaluation and flow cytometric analyses.

There is considerable interest in expanding B cell-targeted therapies for human autoimmune diseases (16–27). A chimeric antibody against human CD20 (hCD20), rituximab, showed limited clinical efficacy in human PBC (28–30). One explanation for its limited efficacy was the appearance of anti-drug antibodies (ADAs; more specifically, human anti-chimeric antibodies, HACAs) as observed in other human autoimmune diseases (31–37). Moreover, HACAs have been associated with adverse events such as serum sickness (38–40). In this study, we studied a novel humanized IgG1 antibody against hCD20 that is less immunogenic compared with chimeric antibodies, enabling intermittent administration with much lower risk of anti-humanized antibody development during treatment of human PBC [Figure 1A; (41)]. This antibody (TKM-011, formerly called BM-ca) has significant direct cytotoxic activity as well as antibody-dependent cell-mediated cytotoxicity (ADCC) activity, similar to rituximab and ofatumumab (42–44). To better reflect human immunobiology, we generated dnTGF-βRII mice expressing hCD20 as well as human Fcγ receptors (hFcγRs) (Figure 1B). After treating these animals with the humanized anti-hCD20 antibody, TKM-011, we observed a significant reduction in portal inflammation, decreased liver-infiltrating mononuclear cells and a reduction in liver CD8^+^ T cells. Importantly, direct correlations were apparent between the numbers of liver non-B cells and B cells and between the numbers of liver memory CD8^+^ T cells and B cells. These effects were less pronounced in mice that developed mouse anti-humanized antibodies (MAHAs). These data suggest that the presence of ADAs limits the effectiveness of B cell-targeted therapies and might be overcome by using fully humanized antibodies.

## Materials and methods

### Generation of hCD20- and hFcγR-expressing IL-10-GFP dnTGF-βRII mice

Human CD20 and FcγR bacterial artificial chromosome (BAC) transgenic mice were generated by the Institute of Immunology Co., Ltd. (Tokyo, Japan) as follows. All *in vivo* experiments and protocols for animal studies were approved by the Laboratory Animal Ethics Committee at Institute of Immunology Co., Ltd. The RP11-792H2 (human) and RP23-117H19 (mouse) BAC clones were selected for construction of a chimeric human-mouse CD20 gene. A hFcγR BAC clone, RP11-925D6, was selected because its 180-kb complete sequence contained the hFcγR gene cluster including the activating FcγRs (FcγR2A, FcγR3A, FcγR2C, and FcγR3) and the inhibitory FcγR2B. A chimeric human-mouse CD20 BAC construct harboring the full-length hCD20 coding region in place of the mouse ortholog was generated by BAC recombineering using the Red/ET Counter Selection BAC Modification Kit (Gene Bridges, Heidelberg, Germany). The human-mouse CD20 BAC and human FcγR BAC constructs were prepared using a Nucleobond Plasmid Purification Kit (MACHEREY-NAGEL, Düren, Germany). For microinjection, both BAC constructs were linearized with PI-*Sce*I endonuclease (New England Biolabs, Beverly, MA). The linearized BAC DNA was separated using pulsed field gel electrophoresis, extracted by electroelution and dialyzed against TE buffer containing 0.1 mM EDTA. The BAC transgenic mice were generated by pronuclear injection of both the hCD20 and hFcγR BAC constructs into C57BL/6J (B6) mouse embryos. Transgenic founders and germline transmission of the BAC transgenic constructs were confirmed by Southern blotting. No obvious gross phenotypic differences were apparent between transgene-positive and -negative littermates. Because hCD20- and hFcγR- expressing mice have functional antibody-dependent cell-mediated cytotoxicity (ADCC) when administered chimeric and humanized anti-hCD20 antibodies (Supplementary Figure 1), several transgenic founders were bred with wild-type B6 mice.

In the next phase of transgenic mouse construction, hCD20- and hFcγR-expressing IL-10-GFP dnTGF-βRII mice were generated as follows. First, dnTGF-βRII mice and IL-10-GFP mice (from The Jackson Laboratory) were bred with B6 mice. We also utilized the IL-10-GFP mice (12, 45, 46) to construct hCD20/hFcγR-expressing IL-10-GFP dnTGF-βRII mice. Briefly, male IL-10-GFP expressing dnTGFβ-RII mice were bred with female hCD20/hFcγR BAC transgenic mice (Supplementary Figure 2) to obtain female heterozygous dnTGFβ-RII mice expressing heterozygous hCD20, hFcγR and IL-10-GFP (hCD20 and hFcγR-expressing IL-10-GFP dnTGF-βRII mice). Of note, heterozygous hCD20 expression was intentionally designed to promote incomplete B-cell depletion when targeting hCD20 (Supplementary Figure 2A). Residual B cells presumably remain after MAHA production against CD20-targeted antibodies allow us to better evaluate the therapeutic efficacy of anti-hCD20 treatment for liver inflammation in the presence or absence of MAHAs. Throughout these studies, only female mice were used and all animals were genotyped to confirm the presence of dnTGF-βRII, hCD20, hFcγR, and IL-10-GFP in genomic DNA at 3–4 weeks of age (45). All mice were fed with sterile standard mouse chow CRF-1 (Charles River Laboratories Japan, Tokyo, Japan) and filtered water and were maintained under specific pathogen-free conditions. This study was performed under a protocol approved by the Institutional Animal Care and Use Committee of Akita University Graduate School of Medicine and Faculty of Medicine.

### Experimental protocol

An optimized dose of the humanized anti-hCD20 monoclonal antibody TKM-011 (250 μg in 250 μL of PBS) or 250 μL of PBS alone was administered intraperitoneally to hCD20- and hFcγR-expressing IL-10-GFP dnTGF-βRII mice using a 26-gauge needle every week starting at 4–6 weeks of age. Peripheral blood samples from individual mice were obtained from the tail vein prior to initial treatment and biweekly thereafter (Figure 1C). Sera were collected and stored at−70°C until use. Cells were used for flow cytometric analyses of peripheral lymphocyte frequency. Mice were sacrificed after 16 weeks of treatment, and tissues (liver, spleen, and colon) and cells were used for pathological and flow cytometric evaluation, respectively.

### ELISA

Serum levels of murine IgG, IgA, and IgM as well as human IgG1 (hIgG1; TKM-011) were measured using mouse IgG, IgA, and IgM quantitative ELISA kits (BETHYL, Montgomery, TX) and a Human IgG1 Ready-SET-Go!™ kit (eBioscience, San Diego, CA), respectively. Standards provided by the manufacturers were used throughout. Serum reactivity of AMAs, anti-TKM-011 antibodies, and anti-infliximab antibodies was quantified using an ELISA Starter Accessory Kit (BETHYL, Montgomery, TX). Briefly, 96-well ELISA plates were coated with either (i) an affinity-purified recombinant fusion protein, pML-MIT-3, which included three distinct lipoyl domains of PDC-E2, the branched-chain 2-oxo-acid dehydrogenase complex, and the 2-oxo glutarate dehydrogenase complex (47), (ii) TKM-011, and (iii) infliximab as an irrelevant control. Briefly, ELISA plated were coated with antigens at 5 μg/mL in the manufacture provided coating buffer at 4°C overnight, washed three times with the manufacture provided washing buffer, and blocked with the manufacture provided blocking buffer for 30 min. Sera (100 μL volume; diluted between 1:200 and 1:40,000) were added to wells of microtiter plates for 1 h at room temperature and the plates were washed again. Horseradish peroxidase-conjugated goat anti-mouse immunoglobulin (100 μL; diluted 1:2,000; Thermo Fisher Scientific, Rockford, IL) was added to each well for 1 h at room temperature, and the wells were washed three times. Immunoreactivity was detected by measuring the optical density (OD) at 450 nm after incubating for 15 min with 100 μL of 3,3′,5,5′–tetramethylbenzidine and the reaction terminated with of 100 μL of 2N sulfuric acid. Previously-calibrated positive and negative standards were included in each assay. An OD ratio of anti-TKM-011 to anti-infliximab reactivity was calculated for each serum sample.

### Flow cytometry

Peripheral blood mononuclear cells (MNCs) were isolated from heparinized murine blood using Histopaque-1.083 (Sigma-Aldrich, Saint Louis, MO) to assess the frequency of B and T cells. Cells were pre-incubated with mouse FcR blocking reagent for 15 min and then incubated at 4°C for 20 min with a pre-determined optimized concentration of PE-conjugated anti-TCR-β (BioLegend) and APC-conjugated anti-CD19 (BioLegend) antibodies. In addition, MNCs were isolated from liver and spleen suspensions as previously described (12, 14, 48). An aliquot of these cells was pre-incubated with FcR blocking reagent and then incubated at 4°C with a combination of fluorochrome-conjugated antibodies, including PE-conjugated anti-CD44 antibody (BioLegend), PE-CF594-conjugated anti-CD4, anti-CD8a and anti-CD19 antibodies (BD biosciences, San Jose, CA), PE-Cy5 conjugated anti-TCR-β antibody (BioLegend), and PE-Cy7-conjugated anti-CD4, anti-CD5, anti-CD8a, and anti-NK1.1 antibodies (BioLegend). Multi-color flow analyses were performed using an Accuri C6 cytometer (BD Biosciences, San Jose, CA) and a Cytomics FC500 flow cytometer (Beckman Courter, Brea, CA) using five-color analysis. The acquired data were analyzed with Accuri C6 cytometer software (BD Biosciences, San Jose, CA) and FlowJo (Ver10.1) software (FlowJo, Ashland, OR).

The following cytokines, chemokines, and growth factors were analyzed. Serum levels of eotaxin, G-CSF, GM-CSF, IFN-γ, IL-1α, IL-1β, IL-2, IL-3, IL-4, IL-5, IL-6, IL-9, IL-10, IL-12(p40), IL-12(p70), IL-13, IL-17A, KC(CXCL-1), MCP-1(MCAP), MIP-1α, MIP-1β, RANTES, and TNF-α were quantified using a Bio-Plex Pro Mouse Cytokine 23-Plex Immunoassay (Bio-Rad, Hercules, CA) according to the manufacturer's protocol. Sera were diluted four or five times and then assayed.

### Tissue pathology and immunohistochemistry

Portions of liver, spleen and colon tissue were excised and immediately fixed with 10% buffered formalin solution for 2 days at room temperature. Paraffin-embedded tissue sections were then cut into 4-μm slices for routine hematoxylin (Merck KGaA, Darmstadt, Germany) and eosin (Muto Pure Chemicals, Tokyo, Japan) (H&E) staining. Scoring of liver inflammation was performed for coded H&E-stained liver sections using a set of six indices by a “blinded” pathologist (KT); the indices included severity and frequency of portal and lobular inflammation (evaluated as 0 = none, 1 = minimal, 2 = mild, 3 = moderate, and 4 = severe inflammation) and bile duct damage (evaluated as 0 = none, 1 = epithelial damage with cytoplasmic change, 2 = epithelial damage with nuclear change, 3 = chronic non-suppurative destructive cholangitis, and 4 = bile duct loss). Frequencies were scored as 1 = none, 2 = 1–10%, 3 = 11–20%, 4 = 20–50%, and 5 = more than 50% frequency. The final scores for portal and lobular inflammation and bile duct damage were calculated as the sum total of the indices for severity and frequency. Liver fibrosis was evaluated as 0 = none, 1 = portal enlargement, 2 = bridging fibrosis, 3 = frequent bridging fibrosis and, 4 = cirrhosis. Liver granuloma was evaluated as 0 = none, 1 = a few, 2 = portal or lobular, 3 = portal and lobular, and 4 = marked. Colon inflammation was evaluated as 0 = none, 1 = minimal, 2 = mild, 3 = moderate, and 4 = severe inflammation (crypt abscess).

Tissue sections were cut at 4-μm thickness from tissue blocks and placed on slides. After de-paraffinization, sections were soaked in a solution of 3% H_2_O_2_ in methanol for 5 min to block endogenous peroxidase, then in target retrieval buffered saline solution (Dako Cytomation, Carpinteria, CA) in a non-metal-containing plastic pressure cooker, and finally heated in a microwave oven for 10 min (maximum 500 W). After heating, sections were washed with PBS for 2 min and then soaked in either a mixture of Mouse on Mouse Blocking Reagent (Vector Laboratories, Burlingame, CA) and mouse FcR Blocking Reagent (BioLegend) or Mouse on Mouse Polymer IHC kit (Abcam; in cases where mouse antibodies were applied) for 3 h. Primary antibodies were diluted to a previously-determined optimized concentration in PBS, applied to the tissue sections in a moist chamber and incubated overnight at room temperature. After three washes with PBS, peroxidase-conjugated polymer Envision kit for mouse primary antibodies (Envision-PO, Envision System, Dako Cytomation) or Histofine-PO for rat primary antibodies (Nichirei, Tokyo, Japan) was applied to the appropriate specimens and incubated for 1 h in the moist chamber. After washing five times with PBS, the sections were immersed in 3,3-diaminobenzidine (DAB) solution (Vector Laboratories), counterstained with hematoxylin (Dako Cytomation) and mounted under coverslips. For double-staining of mouse CD19 (mCD19) and hCD20 only, after detecting mCD19 via the DAB reaction, the sections were soaked in a mixture of Mouse on Mouse Blocking Reagent (Vector Laboratories) and mouse FcR Blocking Reagent (BioLegend) as described above. After PBS washes, anti-hCD20 antibody was applied and incubated overnight in the moist chamber at room temperature. After PBS washes, the Envision kits for mouse and rabbit primary antibodies (Envision-AP, Envision System, Dako Cytomation) were applied and incubated as described above. After PBS washes, the sections were immersed in Fast Red solution (Nichirei).

### Statistical analysis

Values were expressed graphically as means ± standard deviation (SDs). Differences were tested for significance using a two-tailed unpaired Mann-Whitney test and paired Wilcoxon test as appropriate, and associations between paired values were examined using Pearson's correlation and linear regression in Prism (Ver. 7.0) software (GraphPad, La Jolla, CA). Values of *p* < 0.05 were considered statistically significant.

## Results

### Treatment with TKM-011 resulted in reduced frequencies of peripheral B cells, but this effect was attenuated during the latter half of the treatment period after the emergence of anti-TKM-011 antibodies

**Figure 2:**
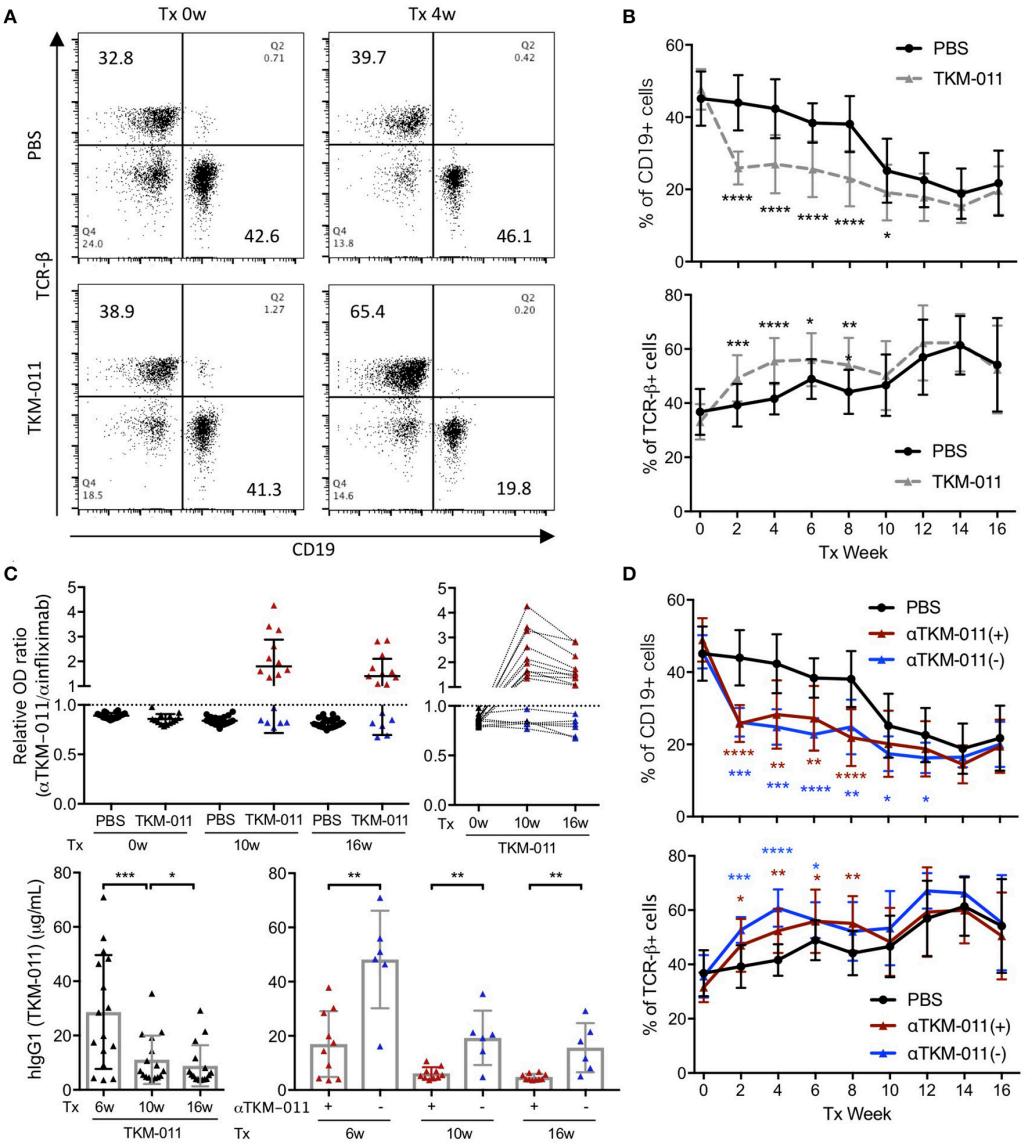
Peripheral B and T cell frequency over the treatment period. **(A)** Representative flow cytometry dot plots prior to the initiation and after 4 weeks of treatment. TKM-011 treatment reduced the frequency of mouse CD19^+^ cells and reversibly increased TCR-β^+^ cells among peripheral mononuclear cells. **(B)** Time course of frequency of CD19^+^ and TCR-β^+^ cells. The frequencies of CD19^+^ B and TCR-β^+^ T cells were significantly lower and higher, respectively, in TKM-011-treated mice compared with controls. **(C)** The ratio of anti-TKM-011:anti-infliximab antibodies was examined in the sera of TKM-011- and PBS-treated mice prior to treatment and at 10 and 16 weeks of treatment. The data are expressed relative to the cut-off value set as 1.0 (mean ± 3 SD of the OD ratio of PBS-treated control mice). The time course of the OD ratio in each TKM-011-treated mouse was also indicated. Serum levels of human IgG1 (hIgG1) were examined in TKM-011-treated mice at 6, 10, and 16 weeks of treatment. TKM-011-treated mice were differentiated by the presence (*n* = 10) or absence (*n* = 6) of anti-TKM-011 antibodies. **(D)** Time course and frequency of CD19^+^ B cells and TCR-β^+^ T cells shown separately in the presence or absence of anti-TKM-011 antibodies. A significantly decreased frequency of CD19^+^ B cells was observed for a longer duration in anti-TKM-011 negative compared with anti-TKM-011 positive mice. [*n* = 20 PBS-treated and *n* = 16 TKM-011-treated mice, with the latter subdivided into *n* = 10 anti-TKM-011 positive mice, shown in red, and *n* = 6 anti-TKM-011 negative mice, shown in blue. *p < 0.05, **p < 0.01, ***p < 0.001, ****p < 0.0001 in Mann-Whitney test and Wilcoxon test for unpaired and paired samples, respectively, in **(B–D)**].

We monitored the frequencies of peripheral CD19^+^ B cells and TCR-β^+^ T cells biweekly over the course of TKM-011 treatment. As shown in Figures 2A,B, the frequency of CD19^+^ B cells decreased by nearly half compared with baseline after 2 weeks of treatment with TKM-011, and were significantly reduced in TKM-011-treated mice during the first half of the TKM-011 treatment period. In contrast, the frequency of peripheral TCR-β^+^ T cells inversely increased in the former half, but the increment was attenuated in the latter half of the TKM-011 treated term. As similar to our previous observation (14), the frequency of peripheral B cells decreased in control mice after 10 weeks of treatment, possibly reflecting intense T cell expansion after 12 weeks of age (unpublished data). Because the increased frequency of peripheral B cells demonstrated lower efficacy of TKM-011 in B-cell depletion during the latter part of treatment (Figure 2B), we examined the presence of anti-TKM-011 antibodies in the sera of TKM-011-treated mice using indirect ELISA. Notably, the relative degree of serum immunoreactivity against TKM-011, compared with infliximab as a control, was significantly elevated after both 10 and 16 weeks of treatment in 10 of 16 TKM-011-treated mice (Figure 2C). Longitudinal data revealed that serum reactivity against TKM-011 persisted in individual mice (Figure 2C). Furthermore, serum levels of hIgG1 (presumably TKM-011) were examined at 6 weeks of treatment, when TKM-011 levels would be expected to be substantially higher, as well as at 10 and 16 weeks. Serum TKM-011 levels were significantly reduced during the latter half of treatment, especially in anti-TKM-011 positive mice (Figure 2C). Significant B-cell depletion was sustained longer in anti-TKM-011 negative mice compared with anti-TKM-011 positive mice (Figure 2D). Taken together, these data suggested that emergence of anti-TKM-011 antibodies was one factor responsible for attenuating B-cell depletion in TKM-011-treated mice.

### TKM-011 treatment affected serum levels of AMAs and immunoglobulins

**Figure 3:**
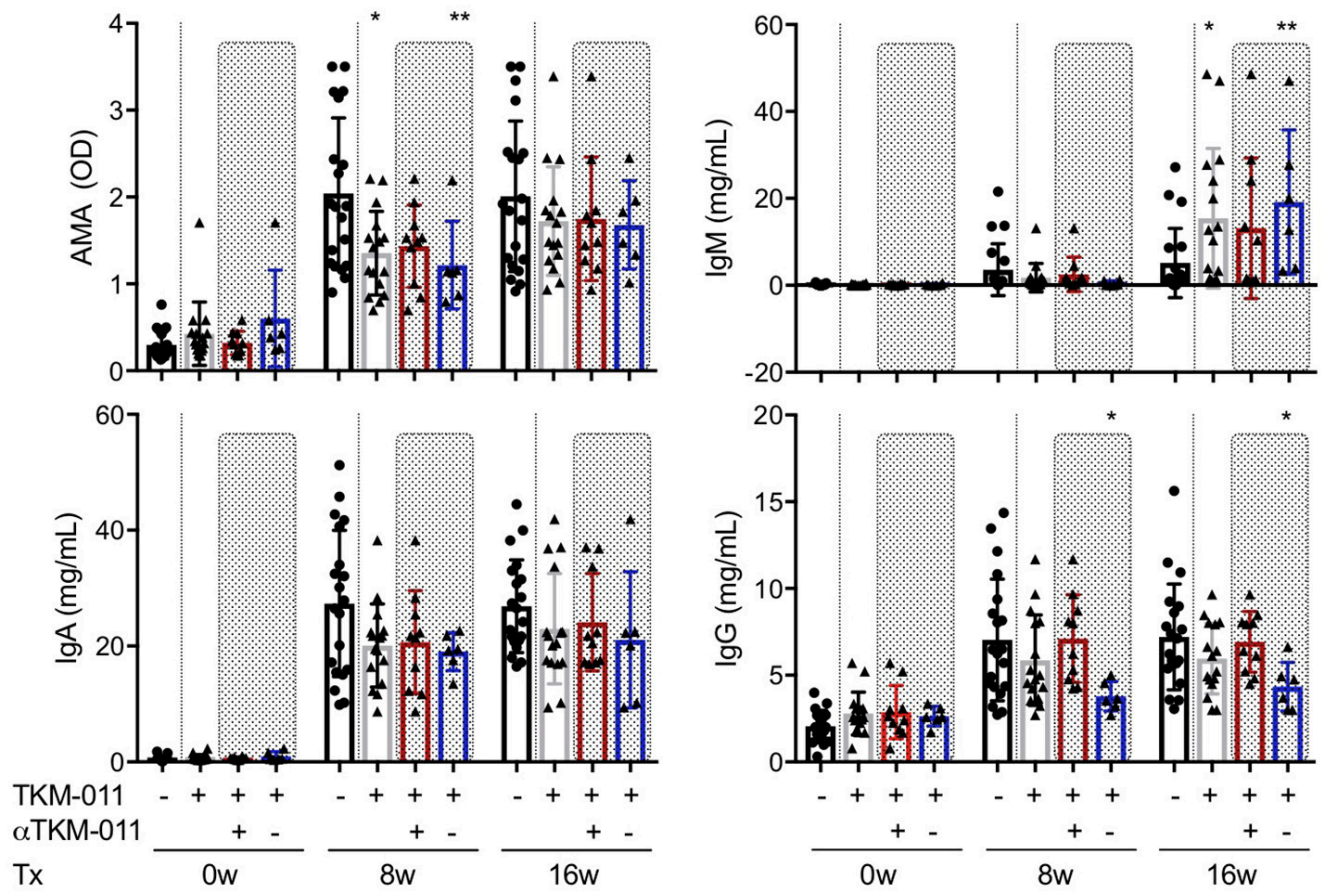
Serum levels of anti-mitochondrial antibodies and immunoglobulins. The titer of anti-mitochondrial antibodies (AMAs) and the concentrations of serum IgM, IgA, and IgG were examined prior to treatment and after 8 and 16 weeks of treatment. Serum AMA reactivity to the recombinant fusion protein MIT-3 was significantly reduced after 8 weeks of TKM-011 treatment, especially in anti-TKM-011 negative mice. Serum levels of IgM were significantly increased in TKM-011-treated mice, especially anti-TKM-011 negative mice, compared with controls after 16 weeks of treatment. Serum levels of IgA were not affected by TKM-011 treatment. Serum levels of IgG were significantly reduced in anti-TKM-011 negative mice compared with PBS-treated control mice (*n* = 20 PBS-treated and *n* = 16 TKM-011-treated mice, with the latter subdivided into *n* = 10 anti-TKM-011 positive mice, shown in red, and *n* = 6 anti-TKM-011 negative mice, shown in blue. *p < 0.05, **p < 0.01 by Mann-Whitney test vs. PBS control).

Because anti-TKM-011 antibodies attenuated B-cell depletion and possibly minimized changes in serum immunoglobulins, we analyzed the serum immunoglobulins of TKM-011-treated mice (anti-TKM-011 positive and anti-TKM-011 negative mice separately), and compared these with PBS-treated controls (Figure 2). The sera of PBS-treated mice contained significant levels of AMAs, which were reduced after 8 weeks of treatment with TKM-011 (Figure 3). There was a significant decrease in serum IgG at 8 and 16 weeks of treatment in anti-TKM-011 negative mice, but an increase in serum IgM at 16 weeks of treatment (Figure 3), suggesting B cell recovery after depletion in TKM-011 treated mice.

### TKM-011 treatment ameliorates liver inflammation in hCD20- and hfcγr-expressing IL-10-GFP dnTGF-βRII mice

**Figure 4:**
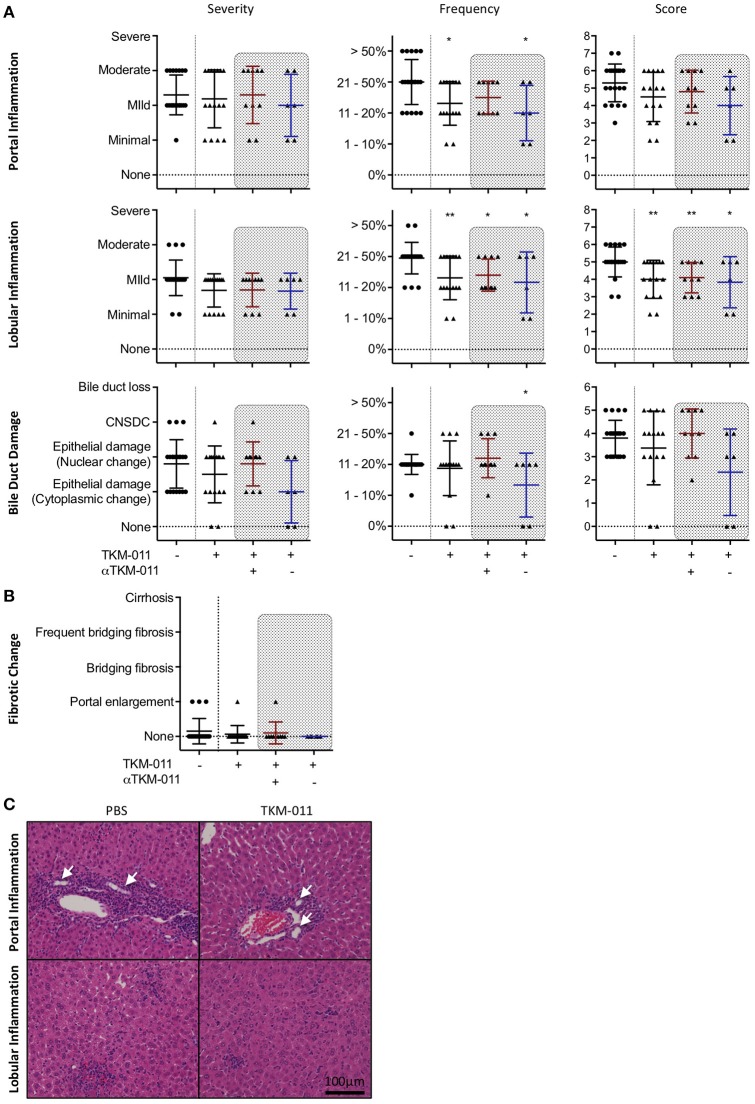
PBC-like liver pathology was ameliorated in TKM-011 treated mice. **(A)** Liver pathological evaluation after 16 weeks of treatment to assess severity, frequency and score in lobular, and portal inflammation as well as bile duct damage. The degree and frequency of portal and lobular inflammation, as well as score for lobular inflammation, was significantly lower in TKM-011 treated mice compared with PBS-treated mice. The frequency of bile duct damage was significantly lower in anti-TKM-011 negative mice. **(B)** Liver fibrotic changes were evaluated after 16 weeks of treatment, but were not observed in anti-TKM-011 negative mice. **(C)** Hematoxylin and eosin (H&E)-stained representative liver sections after 16 weeks of treatment showing that TKM-011-treated mice display milder cellular infiltrates in lobular and portal areas around interlobular bile ducts compared with PBS-treated mice. [*n* = 20 PBS-treated and *n* = 16 TKM-011-treated mice, with the latter subdivided into *n* = 10 anti-TKM-011 positive mice, shown in red, and *n* = 6 anti-TKM-011 negative mice, shown in blue. CNSDC, chronic non-suppurative destructive cholangitis; *p < 0.05, **p < 0.01 by Mann-Whitney test vs. PBS control in **(A)**. In H&E stained sections, a black scale bar indicates 100 μm in **(C)**].

After the 16-week treatment period, liver sections of TKM-011 treated mice exhibited significantly reduced liver inflammation. The degrees of hepatic lobular and portal tract inflammation as well as bile duct damage were plotted individually in Figure 4A. TKM-011 treated mice demonstrated significantly milder portal and lobular inflammation, and 2 of 9 TKM-011 treated mice showed no evidence of bile duct damage. Moreover, anti-TKM-011 negative mice showed significantly less damage to bile ducts, suggesting that TKM-011-induced B-cell depletion efficiently attenuated autoimmune cholangitis in the absence of anti-TKM-011 antibodies. Liver fibrotic changes were observed in 3 of 20 control mice and in 1 of 16 TKM-011 treated mice, but not in any anti-TKM-011 negative mice (Figure 4B). Hepatic inflammatory cell infiltrates were attenuated in TKM-011-treated mice (Figure 4C). No granulomas were observed in either TKM-011-treated or control mice. In a previous study, colitis was exacerbated during treatment with an anti-mouse CD20 antibody that induced total B-cell depletion (14). In this study, colonic inflammation was not induced by the partial B-cell depletion induced by TKM-011 treatment (data not shown).

### Treatment with TKM-011 reduced levels of hepatic CD8^+^ T cell infiltrates in hCD20- and hFcγR-expressing IL-10-GFP dnTGF-βRII mice

**Figure 5:**
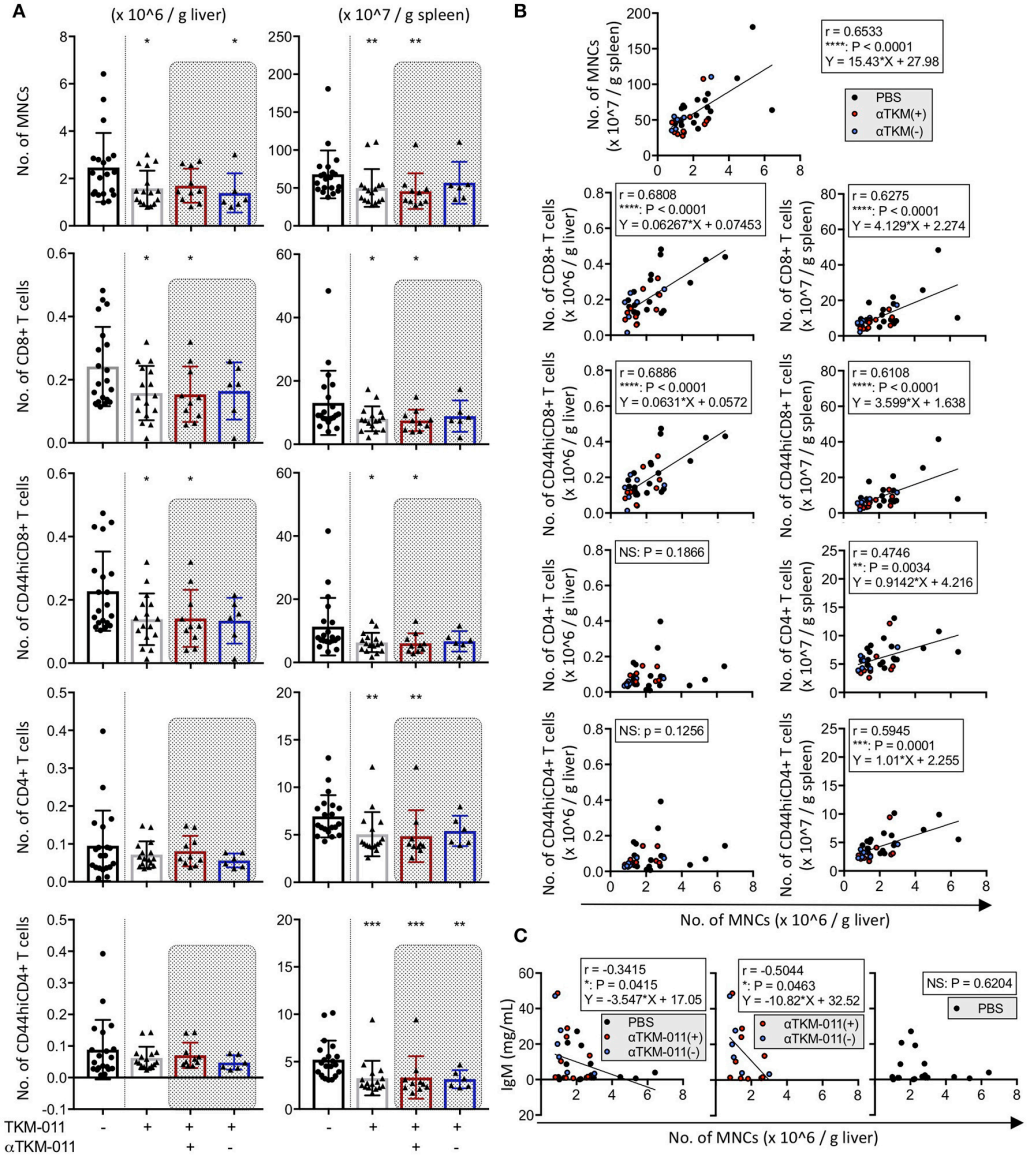
Immunological profiles of hepatic and splenic mononuclear cells (MNCs) in TKM-011-treated mice. **(A)** Absolute numbers of MNCs, CD4^+^ T, CD8^+^ T, CD44^hi^CD4^+^ T, and CD44^hi^CD8^+^ T cells were enumerated per gram of liver and spleen tissue after 16 weeks of treatment. MNC reduction was evident both in liver and spleen of TKM-011-treated mice. Numbers of liver CD8^+^ T cells and their activated subpopulation were significantly decreased in the livers of TKM-011-treated mice along with an obvious reduction in the numbers of CD4^+^ T cells and their activated subpopulation in the spleen after TKM-011 treatment. **(B)** Pearson's correlation between number of liver MNCs with numbers of splenic MNCs, hepatic/splenic CD4^+^ T, CD8^+^ T, CD44^hi^CD4^+^ T, and CD44^hi^CD8^+^ T cells. Linear regression is shown if correlations were apparent. **(C)** Pearson's correlation and linear regression of liver MNC numbers with serum IgM levels. [*n* = 20 PBS-treated and *n* = 16 TKM-011-treated mice, with the latter subdivided into *n* = 10 anti-TKM-011 positive mice, shown in red, and *n* = 6 anti-TKM-011 negative mice, shown in blue. *p < 0.05, **p < 0.01, ***p < 0.001 by Mann-Whitney test vs. PBS control in **(A)**, and for Pearson's correlation analyses in **(B,C)**].

The total number of MNCs both in the liver (1.582 ± 0.7486 vs. 2.465 ± 1.457 × 10^6^/g liver, *p* = 0.0359) and spleen (5.000 ± 2.480 vs. 6.792 ± 3.161 × 10^8^/g spleen, *p* = 0.0091) were significantly reduced after 16 weeks of TKM-011 treatment (Figure 5A). Although the frequency of peripheral B cells was not significantly different after 12 weeks of treatment (Figure 2B), administration of TKM-011 for 16 weeks markedly reduced the total numbers of CD8^+^ T cells (1.577 ± 0.8583 vs. 2.418 ± 1.254 × 10^5^/g liver, *p* = 0.0494; 8.037 ± 3.935 vs. 13.06 ± 10.14 × 10^7^/g spleen, *p* = 0.0422) and numbers of activated CD8^+^ T cells (1.386 ± 0.8159 vs. 2.275 ± 1.252 × 10^5^/g liver, *p* = 0.0298; 6.325 ± 3.066 vs. 11.32 ± 9.095 × 10^7^/g spleen, *p* = 0.0256) in the liver and spleen (Figure 5A). In contrast, the numbers of total and memory CD4^+^ T cells in the liver were unaffected by TKM-011 treatment (7.172 ± 3.522 vs. 9.518 ± 9.298 × 10^4^/g liver, *p* = 0.9937; 6.206 ± 3.558 vs. 8.836 ± 9.414 × 10^4^/g liver, *p* = 0.9875), but were decreased in the spleen (5.064 ± 2.328 vs. 6.947 ± 2.222 × 10^7^/g spleen, *p* = 0.0013; 3.264 ± 1.824 vs. 5.216 ± 2.014 × 10^7^/g spleen, *p* = 0.0001) of TKM-011 treated mice (Figure 5A). Importantly, there was a significant positive correlation between liver MNC numbers and total and memory CD8^+^ T cell numbers (*r* = 0.6806, *p* < 0.0001; *r* = 0.6886, *p* < 0.0001), splenic MNC numbers (*r* = 0.6533, *p* < 0.0001), total and memory CD4^+^ T cell numbers (*r* = 0.4746, *p* = 0.0034; *r* = 0.5945, *p* = 0.0001), as well as total and memory CD8^+^ T cell numbers (*r* = 0.6275, *p* < 0.0001; *r* = 0.6108, *p* < 0.0001; Figure 5B). These findings suggested that liver CD8^+^ T cells were not the only mediators of the liver pathology observed in this mouse model of PBC, in agreement with a previous report (49), and that both CD4^+^ and CD8^+^ T cells in the spleen contribute to liver inflammation. In contrast, the changes in number of hepatic CD4^+^ T cells did not correlate with the changes in number of liver MNCs. Associations between liver MNC numbers and serum levels of immunoglobulins as well as of hIgG1 (TKM-011) were also assessed. IgM levels after 16 weeks of TKM-011 treatment were negatively correlated with liver MNC numbers (*r* = −0.3415, *p* = 0.0415; Figure 5C); no other serum constituent (i.e., AMAs, IgA, IgG, or hIgG1) was significantly correlated with MNC numbers (data not shown). The inverse correlation between IgM levels and liver MNC frequency was apparent in TKM-011-treated mice (*r* = −0.5044, *p* = 0.0463), but not in control mice.

Due to the minimal effect of TGF-β signaling on T and NKT cells in this mouse model of PBC (12), the immunoregulatory function of IL-10 could instead potentially attenuate liver inflammation. We therefore examined the absolute numbers of IL-10-GFP positive CD4^+^ T cells in the liver and the spleen, but these were comparable in TKM-011-treated and control mice (data not shown).

### Treatment with TKM-011 affected KC, IL-12p40, and IL-5 levels in sera of hCD20- and hFcγR-expressing IL-10-GFP dnTGF-βRII mice

**Figure 6:**
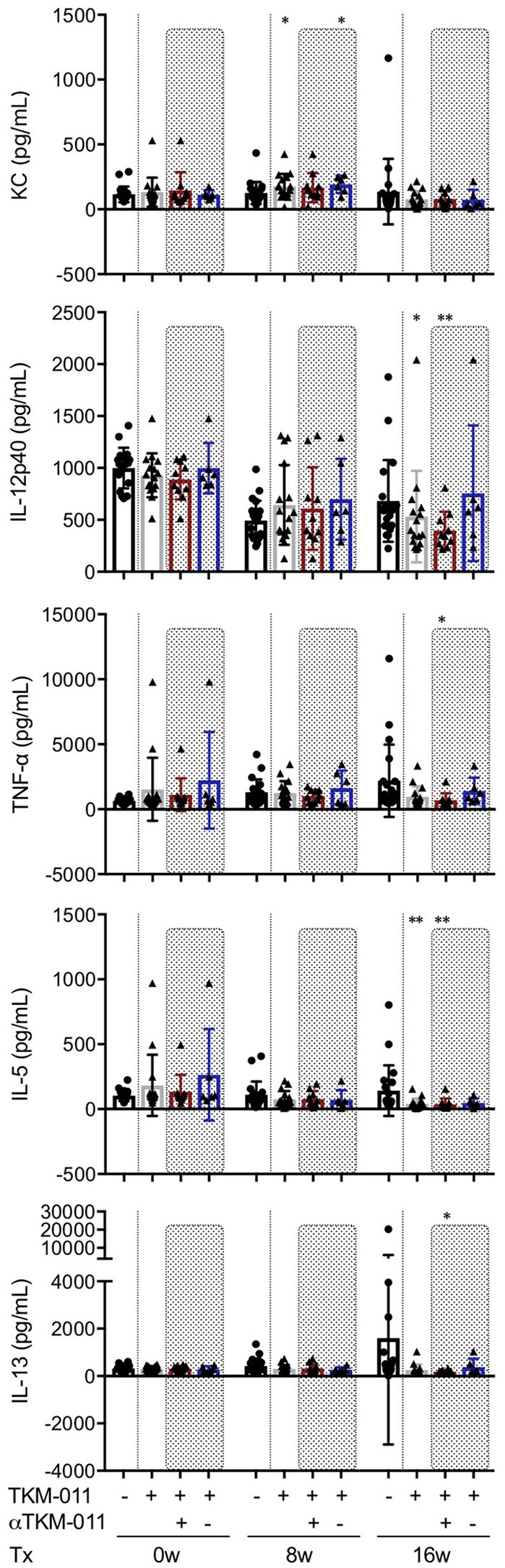
Serum cytokine levels during TKM-011 treatment. Mean serum cytokine levels were measured prior to treatment initiation and at 8 and 16 weeks of TKM-011 treatment using a Bio-Plex Pro Mouse Cytokine 23-Plex Immunoassay. Keratinocyte-derived chemokine (KC) was significantly increased in TKM-011 treated mice compared with controls after 8 weeks of treatment. TKM-011 treatment reduced IL-12p40 and IL-5 levels after 16 weeks of treatment, whereas TNF-α and IL-13 levels were decreased in anti-TKM-011 positive mice (*n* = 20 PBS-treated and *n* = 16 TKM-011-treated mice, with the latter subdivided into *n* = 10 anti-TKM-011 positive mice, shown in red, and *n* = 6 anti-TKM-011 negative mice, shown in blue. *p < 0.05, **p < 0.01 by Mann-Whitney Test vs. PBS control).

Serum levels of pro- and anti-inflammatory cytokines and chemokines were examined extensively throughout the TKM-011 treatment period. Mouse keratinocyte-derived chemokine (KC, also known as the mouse IL-8 analog, CXCL1) is generally thought to be a pro-inflammatory chemokine, but may have an anti-inflammatory effect on liver inflammation in this model and other murine models of other autoimmune diseases (50, 51). Serum levels of CXCL1/KC were higher after 8 weeks of TKM-011 treatment compared with those of PBS-treated mice (Figure 6). TKM-011-treated mice also demonstrated a significant reduction in levels of the inflammatory cytokine, IL-12p40. Moreover, levels of the B-cell and eosinophil-stimulating cytokine, IL-5 were decreased after 16 weeks of TKM-011 treatment (Figure 6). Significantly reduced levels of TNF-α and IL-13 were observed only in anti-TKM-011 positive mice (Figure 6). None of the other cytokines and chemokines were affected by TKM-011 treatment (data not shown).

### The immunohistochemical profiles of B cells, hFcγR-expressing cells, and higG1-loaded cells during anti-TKM-011 antibody emergence

**Figure 7:**
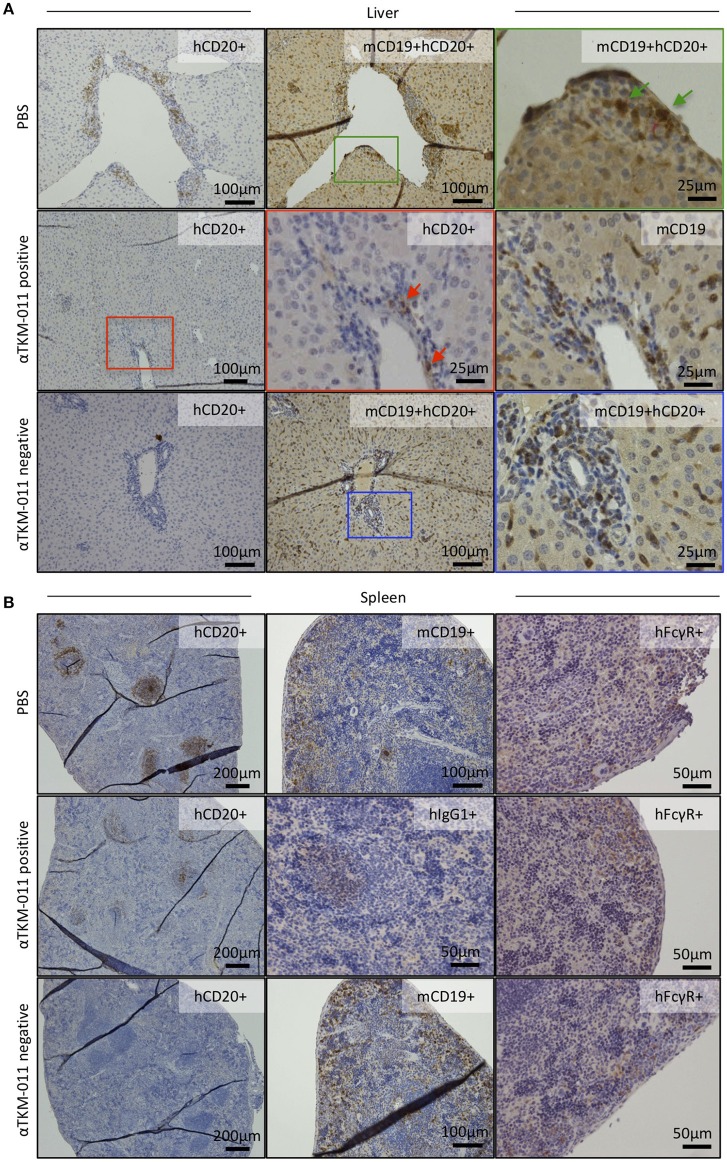
Generation of anti-TKM-011 antibodies leads to incomplete depletion of hCD20-expressing cells. Representative liver and spleen sections of PBS-treated and TKM-011-treated anti-TKM-011 positive and negative mice. Staining for human CD20^+^ (hCD20^+^), mouse CD19^+^ (mCD19^+^), hCD20^+^mCD19^+^ double-positive, human IgG1^+^ (hIgG1^+^), and human FcγR^+^ (hFcγR^+^) cells was performed. **(A)** Double-positive hCD20^+^mCD19^+^ cells detected in PBS-treated mouse liver were indicated with green arrows, whereas no double-positive cells were observed in the livers of anti-TKM-011 negative mice. Representative TKM-011-treated, anti-TKM-011 positive mice demonstrated hCD20^+^ cells in the portal area whereas CD19^+^ cells were numerously found in a serial liver section (red arrows indicate hCD20^+^ cells). **(B)** In spleens of anti-TKM-011 positive mice, hCD20^+^ and hIgG1^+^ (i.e., TKM-011^+^) cells were also detected, whereas hFcγR expression was the same in all groups.

Seeing that anti-TKM-011 antibodies were sustained during TKM-011 treatment (Figure 2C), we immunohistochemically examined B cells, hFcγR-expressing cells, and hIgG1-loaded cells in the liver and spleen of mice that were positive or negative for anti-TKM-011 antibodies (Figures 7A,B). Singly mCD19^+^ and hFcγR^+^ cells were invariably observed (Figures 7A,B), whereas liver hCD20^+^mCD19^+^ double-positive cells were observed in PBS-treated mice but not in anti-TKM-011 negative mice (Figure 7A). On the other hand, fewer hCD20^+^ cells were observed in the livers and spleens of anti-TKM-011 positive mice (Figure 7A), whereas hIgG1^+^ cells were evident (Figure 7B). This hIgG1^+^ cell population was not observed in anti-TKM-011 negative mice.

### B cells are crucial directors of non-B cell and CD8^+^ T cell liver infiltrates in hCD20- and hFcγR-expressing IL-10-GFP dnTGF-βRII mice

**Figure 8:**
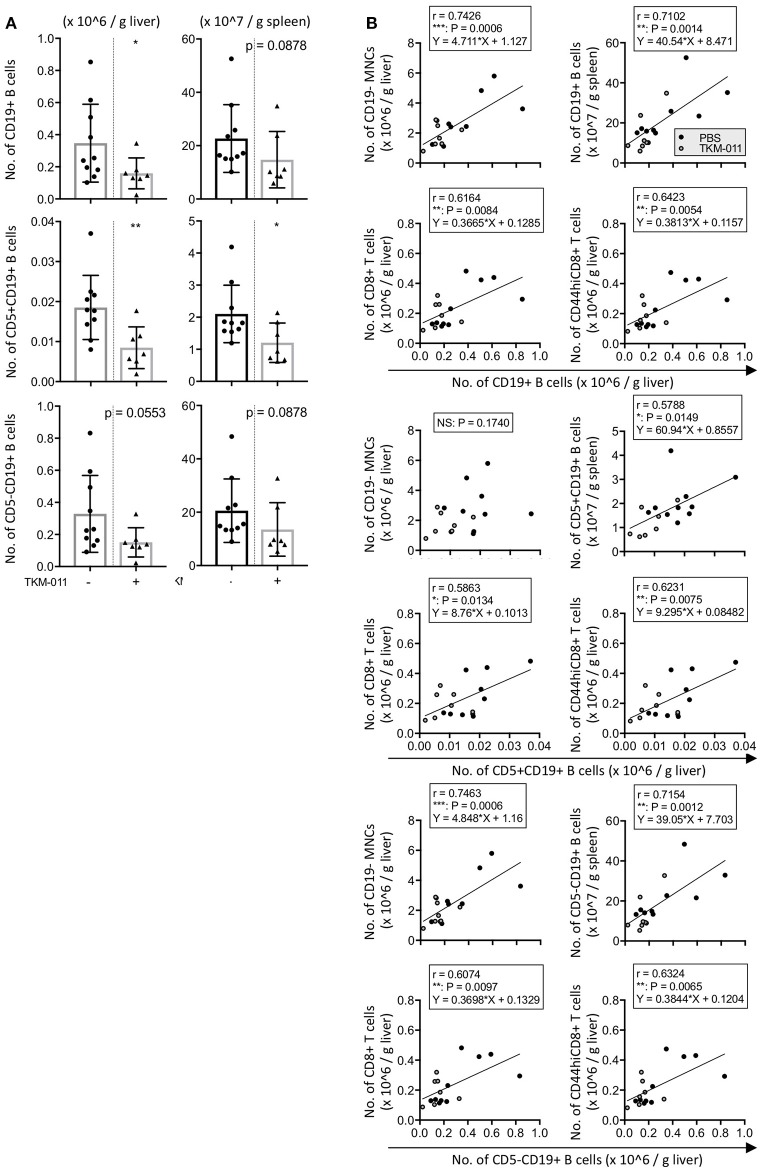
TKM-011 induced a reduction of liver B cells that was significantly correlated with CD19^−^ liver cellular infiltrates. **(A)** Total B cells in the liver and CD5^+^ B cells in the liver and spleen were significantly reduced after 16 weeks of TKM-011 treatment. **(B)** Pearson's correlations between liver total CD19^+^, CD5^+^CD19^+^, and CD5^−^CD19^+^ B cells and liver CD19^−^ MNCs, CD8^+^ T, and CD44^hi^CD8^+^ T cells, as well as splenic total CD19^+^, CD5^+^CD19^+^, and CD5^−^CD19^+^ B cells were examined. Linear regression was shown if a correlation was apparent. Liver total and CD5^−^ B cell numbers were positively correlated with numbers of liver CD19^−^ MNCs and total and memory CD8^+^ T cells, while liver CD5^+^ B cell numbers were correlated with total and memory CD8^+^ T cell numbers. [*n* = 10 and 7 for PBS- and TKM-011-treated mice, respectively. *p < 0.05, **p < 0.01 by Mann-Whitney test in **(A)**, and for Pearson's correlation analyses in **(B)**].

Because hCD20^+^ cells were observed in the liver and spleen of anti-TKM-011 positive mice, we next examined the numbers of hepatic and splenic B cells as well as associations between hepatic B cells and liver inflammatory cell infiltrates and between hepatic B cells and splenic B cells. In TKM-011-treated mice, absolute numbers of CD19^+^ B cells were significantly reduced in the liver (1.594 ± 0.961 vs. 3.471 ± 2.426 × 10^5^/g liver, *p* = 0.0431) but not in the spleen (1.475 ± 1.058 vs. 2.267 ± 1.269 × 10^8^/g spleen, *p* = 0.0878; Figure 8A). TKM-011-treated mice also showed a trend toward decreased CD5^+^CD19^+^ B cells in the liver and spleen (0.8471 ± 0.5212 vs. 1.853 ± 0.8007 × 10^4^/g liver, *p* = 0.0074; 1.204 ± 0.6144 vs. 2.102 ± 0.8967 × 10^7^/g spleen, *p* = 0.0431), although this did not achieve statistical significance for CD5^−^CD19^+^ B cells (1.509 ± 0.9114 vs. 3.286 ± 2.396 × 10^5^/g liver, *p* = 0.0553: 1.354 ± 1.001 vs. 2.057 ± 1.188 × 10^8^/g spleen, *p* = 0.0878). We also examined the absolute numbers of hepatic and splenic IL-10-GFP positive B cells: these were not detectable in the liver, and their numbers in the spleen were comparable between TKM-011-treated and control mice (data not shown). Importantly, numbers of liver CD19^+^ B cells was positively correlated with numbers of liver CD19^−^ MNCs (*r* = 0.7426, *p* = 0.0006), liver total and memory CD8^+^ T cells (*r* = 0.6164, *p* = 0.0084; *r* = 0.6423, *p* = 0.0054), and splenic CD19^+^ B cells (*r* = 0.7102, *p* = 0.0014; Figure 8B), suggesting that B cells are crucial mediators of liver inflammation and that B-cell depletion using anti-CD20 antibodies ameliorates autoimmune cholangitis.

## Discussion

The significance of B cell in the immunopathology of autoimmune cholangitis is well documented in patients with PBC and animal models (6, 9, 10, 13, 15, 52–55). This study demonstrated that B cells promote the development of PBC-like liver disease, and partial but substantial B-cell depletion with humanized anti-hCD20 antibody (TKM-011) effectively attenuates bile duct damage in the hCD20 hFcγR-expressing dnTGF-βRII mice. Specifically, we administered TKM-011 intraperitoneally weekly for 16 weeks to target peritoneal cavity B cells as peripheral B cells (14, 56). Because hCD20 expression is limited to 47.2 ± 4.6% of mCD19^+^ B cells in hCD20-heterozygous mice (Supplementary Figure 2A), peripheral B cells were significantly depleted 2 weeks after initial treatment with TKM-011, but still retained at approximately 50% of the initial frequency. Despite the limited B-cell depletion, TKM-011 treatment reduced the numbers of MNCs as well as total and memory CD8^+^ T cells both in the liver and spleen. Splenic CD4^+^ T cells were also reduced in TKM-011-treated mice. Moreover, our data demonstrated significant positive correlations between numbers of liver CD8^+^ T cell populations with number of liver MNCs. Using adoptive transfer, we previously demonstrated that CD8^+^ T cells were the primary effectors for autoimmune cholangitis in the dnTGF-βRII mice (49, 57, 58) and were predominantly accumulate in the portal area (49). Also, we previously observed a dramatic decrease in liver CD8^+^ T cells as well as a non-inflamed liver pathology in 5 out of 7 mice after a B cell depletion using anti-mouse CD20 antibody (14). In this study, we observed significant reduction in liver inflammation, and we noted that the number of hepatic CD8^+^ T cells positively correlated with the numbers of liver MNCs as well as B cells, including CD5^+^ and CD5^−^ B-cell subsets. Majority of CD5^+^ B cells normally reside in peritoneal cavity and are poorly susceptible for CD20-targeted B cell depleting treatment whereas splenic CD5^+^ B cells are moderately susceptible but less than other mature, T1, T2 and marginal zone B cells (56). In this study, a 16-weeks anti-CD20 weekly treatment significantly decreased CD5^+^ B cells similarly in the liver and spleen. The reduction in hepatic CD8^+^ T cells further suggests that incomplete but substantial B-cell depletion still effectively attenuates hepatic CD8^+^ T cell infiltrates in this mouse model. CD8^+^ T cells proliferate to a greater extent in the presence of B cells activated by anti-Ig and anti-CD40 compared with anti-CD3 stimulation alone (59). CD40 ligand-stimulated B cells were able to potently present antigens directly to naïve T cells *in vivo*, generating CD8^+^ effector cells that secreted proinflammatory cytokines and damaged target cells (60).

In autoimmune diabetic mice expressing hCD20, transient treatment with a murine anti-hCD20 antibody delayed disease onset, significantly depleted B cells, and reduced the frequency of CD44^+^CD8^+^ memory T cells as well as the frequency of repopulating B cells that express the co-stimulatory molecules CD80/86 upon anti-CD40 stimulation (61). Taken together, our data suggest that limited B cell depletion may sufficiently suppress the development and expansion of mitochondrial autoantigen-specific CD8^+^ T cells in this murine model of PBC (58).

The magnitude of peripheral B-cell depletion was highly significant during the middle of the treatment period but dwindled toward its end. This observation can be accountable by B cell recovery process in TKM-011-treated mice. In addition, serum IgM levels decreased slightly during TKM-011 treatment, but were significantly elevated after 16 weeks of treatment. Although the level of serological IgM levels after 16 weeks of treatment were negatively correlated with liver MNC numbers in TKM-011 treated mice, the number of liver B cells was positively correlated with liver non-B and CD8^+^ T cells. Thus, it is unlikely that B-cell repopulation and/or proliferation occurred within the liver.

We have previously observed elevated serum pro-inflammatory cytokines in the dnTGF-βRII mouse model of PBC (14, 52, 57). Here, we conducted a longitudinal analysis of an extensive panel of serum cytokines and chemokines in TKM-011-treated mice and control mice over the duration of treatment. In this study, serum levels of KC significantly increased during TKM-011 treatment (Figure 6). Overexpression and administration of recombinant CXCL1/KC suppresses murine autoimmune diseases such as experimental autoimmune encephalomyelitis (51) and autoimmune myocarditis; in the latter case treatment also reduced the numbers of autoreactive effector T cells specific to cardiac self-peptides (50). CXCL1/KC (also known as GRO-α) is a chemotactic factor for B cells (62). B cells of younger individuals express higher levels of CXCL1/KC when subjected to an inflammatory environment with LPS (63). Moreover, intra-allograft B cells cultivated *in vitro* can potently produce CXCL1/KC during chronic allograft damage after renal transplantation (64). These reports suggest that KC released from inflammatory sites acts as not only an immunosuppressive agent but also an enhancer of B-cell migration to inflammatory sites and as a signal for migrant B cells to produce more CXCL1/KC.

IL-12p40 is one of the pro-inflammatory cytokines that was elevated in the dnTGF-βRII mice (12) and depletion of IL-12p40 significantly ameliorated liver inflammation accompanied with a reduction of the Th1 cytokine, TNF-α (65). In TKM-011 treated mice, the level of serum IL-12p40 levels was markedly reduced upon prolonged TKM-011 treatment at 16 weeks. Decreased levels of a Th2 cytokine, IL-5, were also observed. In human PBC, the presence of IL-5 is associated with eosinophilic cytotoxicity in portal areas (66), and its secretion was significantly enhanced upon *in vitro* T-cell receptor stimulation of CD8^+^CD57^+^ T cells; the frequency of the CD45RO^high^ subpopulation of these cells was increased in early-stage PBC (67). In mice, IL-5 is as effective as IL-2 in promoting cytotoxic T cell (CTL) responses, and both CD8^+^ and CD4^+^ T cells in the spleen can produce IL-5 as well as IL-2, TNF-α, and IFN-γ upon stimulation with specific antigen (68). In the absence of IL-5, the frequency of CTL progenitors was reduced in the spleens of immunized mice (68). Thus, B-cell depletion upon TKM-011 treatment induced a decrease in both hepatic and splenic CD8^+^ T cells as well as CD4^+^ T cells that led to a decrease in serum IL-5, possibly inducing a delay in hepatic CD8^+^ T-cell expansion.

Another Th2 cytokine, IL-13, exerts a pro-fibrotic effect on infectious and autoimmune liver inflammation (69–71); however, no significant reduction of fibrotic changes was observed in TKM-011-treated mice (Figure 4B). Taken together, upon continuous weekly treatment of TKM-011, decreased levels of both IL-5 and IL-13 became evident and likely contributed to the disease attenuation process in these animals. Our previous work have demonstrated that depletion of B cells with anti-mouse CD20 resulted in significant elevation in IL-6, amelioration of liver inflammation but exacerbation of colon inflammation (14). In contrast, IL-6 depletion exacerbated autoimmune cholangitis in conjunction with hepatic B cell and CD8^+^ T cell accrual, but ameliorated inflammatory bowel disease (72). In this study, TKM-011 induced partial B-cell depletion which attenuated autoimmune cholangitis, but neither affected the level of serum IL-6 nor colon inflammation, suggesting that the degree of liver inflammation was positively correlated with number of hepatic B cells as long as IL-6 expression was stable.

IL-10-producing T and B cells, as well as serum levels of IL-10, were similar in TKM-011-treated and control mice (data not shown). Thus, it is unlikely that T cell-derived IL-10 play any immune-regulatory function in the suppression of autoimmune cholangitis after partial B-cell depletion using TKM-011.

The generation of ADAs is a critical immunopharmacological concern in biologics therapeutics. ADA can form immune complex, which further amplify ADA formation and hence poses significant clinical consequences (73). The administration of therapeutic TKM-011 antibody induced the development of anti-TKM-011 antibodies in some mice and affected the serum levels of TKM-011. We could not find any possible factors resulting in non-development of anti-TKM-011 antibodies in the other mice. However, in this study, we did not attempt to dissociate the immunocomplexes of anti-TKM-011 antibodies with TKM-011 prior to our ELISA assay when we assayed for serum levels of anti-TKM-011. This could have under-estimated the actual amount of anti-TKM-011 antibodies in the samples. Although a low titer of anti-TKM-011 antibodies may be present in anti-TKM-011 negative mice, hCD20^+^mCD19^+^ double-positive cells were not detected in either the liver or spleen of anti-TKM-011 negative mice but hCD20^+^ cells were detected in anti-TKM-011 positive mice. Because hFcγR^+^ cells were observed in anti-TKM-011 positive mice at similar levels in negative and control mice, anti-TKM-011 antibodies might prevent hFcγR-expressing cells from binding TKM-011 (hIgG1) on hCD20^+^ cells, resulting in insufficient depletion of hCD20^+^ B cells (Supplementary Figure 3).

In a pilot study in which rituximab given to PBC patients who insufficiently responded to ursodeoxycholic acid, there was a significant reduction of serum alkaline phosphatase (ALP), AMA and IgM levels in some patients (29). Attenuation of pruritus with rituximab was also observed in another PBC cohort (28). On the other hand, other studies indicated that rituximab was not effective in the management of fatigue (74) but could transiently reduce or normalize the serum levels of ALP in PBC patients (30). However, as a chimeric antibody, rituximab carries the risk of inducing human anti-chimeric antibodies (HACA) compared with humanized antibodies (41). HACAs can impair B cell functions and decrease its efficacy in human autoimmune disease treatments (31–36). Indeed, the emergence of HACAs has been reported in three of 13 PBC patients within 12 months of a two-dose rituximab infusion, 2 weeks apart (28). Our results suggest that conformational epitopes are immunogenic both in the variable regions as well as in the constant regions of hIgG1 in mice. Considering that the chimeric antibody rituximab has been used in clinical studies for patients with PBC (28–30), we also examined rituximab treatment using the same protocol as TKM-011 in this PBC mouse model. Although rituximab would be presumably less immunogenic in mice compared with the humanized antibody TKM-011, anti-rituximab antibodies developed in 6 out of 7 rituximab-treated mice and no pathological improvement was observed (Supplementary Figure 4). In addition, treatment with lower doses (50 μg in 250 μL of PBS) of either TKM-011 or rituximab was ineffective in ameliorating liver inflammation (data not shown). Thus, in contrast with rituximab, TKM-011 treatment attenuated autoimmune cholangitis in this mouse model of PBC. In humans, the mouse Ig variable regions in chimeric antibodies are substantially immunogenic and can generate ADAs (41). Therefore, humanized antibodies have an advantage over chimeric antibodies. Optimization of dosage and therapeutic protocols including intermittent administration in clinical trials are needed for more comprehensive assessment of humanized antibodies in autoimmune diseases therapy.

## Author contributions

YM, ASh, and MG contributed conception and designed the study. YM, KT, YN, and KK contributed to data collection. YM performed the statistical analysis. YM and MG wrote the manuscript. YO, Z-XL, WZ, G-XY, SU, MT, AO, TS, ASa, DW, MM, YU, PL, AT, and MH interpreted the results and contributed to manuscript revision. All authors read and approved the submitted manuscript.

### Conflict of interest statement

The authors declare that the research was conducted in the absence of any commercial or financial relationships that could be construed as a potential conflict of interest.

## Supplementary material

Supplementary Figure 1 Splenic B-cell depletion in human CD20 expressing BALB/c mice in the presence or absence of human FcγR expression. **(A,B)** Representative flow cytometry dot plots. Splenic mononuclear cells (MNCs) were pre-incubated with mouse FcR blocking reagent and then incubated at 4°C with a combination of fluorochrome-conjugated antibodies (BD Biosciences), including FITC-conjugated anti-CD45R/B220, and PE-conjugated anti-human CD20 (hCD20) as well as FITC-conjugated anti-mouse CD49b/DX5 and PE-conjugated anti-human CD16 (hCD16, hFcγRIII). Cell-surface expression of hCD20 in CD45R^+^ B cells was observed in hCD20-expressing mice regardless of hFcγR expression. Cell-surface expression of hCD16 in CD49b^+^ NK cells was observed in hCD20- and hFcγR-expressing mice, but not in hFcγR-negative mice. **(C)** Anti-hCD20 humanized antibody TKM-011 (250 μg in 250 μL of PBS) and the chimeric antibody rituximab (250 μg in 250 μL of PBS) or 250 μL of PBS alone (as a control) were injected intraperitoneally into hCD20-expressing BALB/c mice in the presence or absence of hFcγR expression. Spleens were extracted 7 days after the injection. Splenic MNCs were counted, and an aliquot of these cells was stained as shown above and analyzed using flow cytometry. Absolute numbers of total CD19^+^ cells were calculated. Enhanced B-cell depletion was observed in mice expressing both hCD20 and hFcγR, suggesting an *in vivo* functional mechanism of hFcγR in mediating antibody-dependent cell-mediated cytotoxicity (ADCC).

Supplementary Figure 2 Human CD20 and FcγR-expressing B6 mice. Splenic mononuclear cells were pre-incubated with mouse FcR blocking reagent and then incubated at 4°C with a combination of fluorochrome-conjugated antibodies (BD Biosciences), including APC-conjugated anti-mouse CD19 and PE-conjugated anti-human CD20 as well as FITC-conjugated anti-CD49b/DX5 and PE-conjugated anti-human CD16 (hCD16, hFcγRIII). Cells were analyzed using flow cytometry. **(A)** Cell-surface expression of hCD20 was observed in 47.2% of CD19^+^ B cells. **(B)** Cell-surface expression of hCD16 was also observed in CD49b^+^ NK cells.

Supplementary Figure 3 Graphical abstract. Anti-drug antibody against a novel humanized anti-CD20 antibody impair its therapeutic effect on primary biliary cholangitis in human CD20- and FcγR-expressing mice.

Supplementary Figure 4 Rituximab treatment did not ameliorate liver pathology. Rituximab was administered using the same protocol as TKM-011 treatment in the mouse model of PBC. **(A)** Anti-rituximab antibodies were observed in 6 of 7 treated mice. Serum levels of hIgG1 were gradually reduced over the course of treatment. **(B)** Frequencies of CD19^+^ and TCR-β^+^ cells were transiently reduced and increased, respectively, in rituximab-treated mice. **(C)** Rituximab treatment did not improve liver inflammation or bile duct damage after 16 weeks of treatment (*n* = 20 and 7 for PBS- and rituximab-treated mice, with the latter subdivided into *n* = 6 anti-rituximab antibody positive mice, shown in red, and *n* = 1 anti-rituximab antibody negative mouse, shown in blue. CNSDC, chronic non-suppurative destructive cholangitis; ^*^*p* < 0.05, ^**^*p* < 0.01, ^***^*p* < 0.001, ^****^*p* < 0.0001 by Mann-Whitney Test vs. PBS control and Wilcoxon Test for paired samples).

## Abbreviations

Ab: antibody
ADA: anti-drug antibody
AMA: anti-mitochondrial antibody
ADCC: antibody-dependent cell-mediated cytotoxicity
CDC: complement-dependent cytotoxicity
CXCL1/KC: chemokine (C-X-C motif) ligand 1/keratinocyte-derived chemokine
dnTGF-βRII mice: TGF-β receptor II dominant negative mice
HACA: human anti-chimeric antibody
hCD20: human CD20
hFcγR: human Fcγ receptor
IL: interleukin
MNC: mononuclear cell
PBC: primary biliary cholangitis
TGF-β: transforming growth factor-β
TNF-α: tumor necrosis factor-α.
