# Supplementary material for: Anti-drug Antibodies Against a Novel Humanized Anti-CD20 Antibody Impair Its Therapeutic Effect on Primary Biliary Cholangitis in Human CD20- and FcγR-Expressing Mice
Source: Front Immunol. 2018 Nov 2;9:2534. doi: 10.3389/fimmu.2018.02534 (PMC6224429; doi:10.3389/fimmu.2018.02534)

Supplementary Materials

Original article:

Anti-drug antibodies against a novel humanized anti-CD20 antibody impair its therapeutic effect on primary biliary cholangitis in human CD20- and FcγR-expressing mice

Authors Names:

Yuki Moritoki[1,2,3], Koichi Tsuneyama[4], Yuka Nakamura[1], Kentaro Kikuchi[5], Akira Shiota[6], Yoshiyuki Ohsugi[7], Zhe-Xiong Lian[8], Weici Zhang[9], Guo-Xiang Yang[9], Shigeharu Ueki[1], Masahide Takeda[1], Ayumi Omokawa[1], Tomoo Saga[1], Akiko Saga[1], Daisuke Watanabe[10], Masahito Miura[11], Yoshiyuki Ueno[12], Patrick S.C. Leung[9], Atsushi Tanaka[13], M. Eric Gershwin[9], and Makoto Hirokawa[1]

Affiliations:

[1]Department of General Internal Medicine and Clinical Laboratory Medicine, Akita University Graduate School of Medicine, Akita 010-8543, Japan

[2]Center for Medical Education and Training, Akita University Hospital, Akita 010-8543, Japan

[3]SimTiki Simulation Center, John A. Burns School of Medicine, University of Hawaii, Honolulu 96813-5534, USA.

[4]Department of Pathology & Laboratory Medicine,

Institute of Biomedical Science, Tokushima University Graduate School of Medicine, Tokushima 770-8503, Japan

[5]Department of Fourth Internal Medicine, Teikyo University Mizonokuchi Hospital, Kawasaki 213-0001, Japan

[6]Institute of Immunology, Co., Ltd. Tokyo 112-0004, Japan

[7]Ohsugi BioPharma Consulting, Co., Ltd. Tokyo 111-0032, Japan

[8]Chronic Disease Laboratory, Institutes for Life Sciences and School of Medicine, South China University of Technology, Guangzhou 510006, China

[9]Division of Rheumatology, Allergy and Clinical Immunology, Genome and Biomedical Sciences Facility, University of California at Davis, Davis, CA 95616, USA

[10]Watanabe Internal Medicine Clinic, Noshiro 016-0856, Japan

[11]Department of Gastroenterology, Omagari Kosei Medical Center, Omagari 014-0027, Japan

[12]Department of Gastroenterology, Yamagata University Faculty of Medicine, Yamagata 990-9585, Japan

[13]Department of Medicine, Teikyo University School of Medicine, Tokyo 173-0003, Japan

Supplementary Figure 1

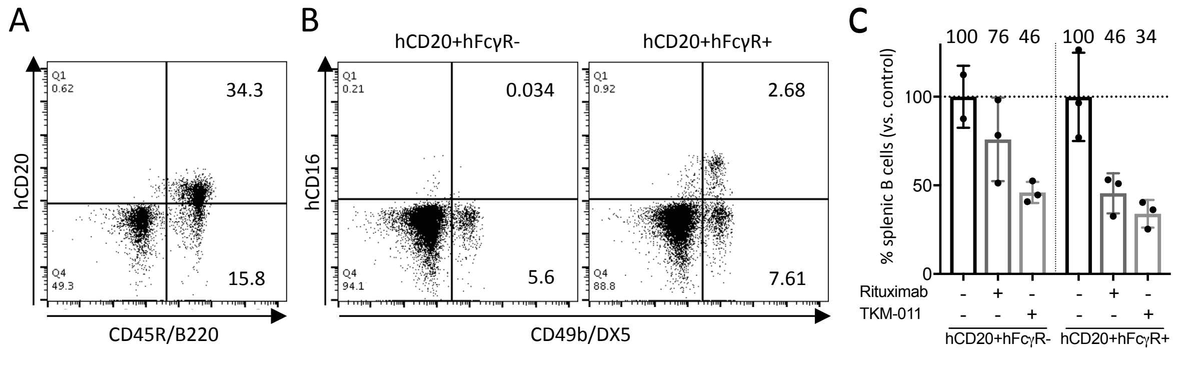

Supplementary Figure 2

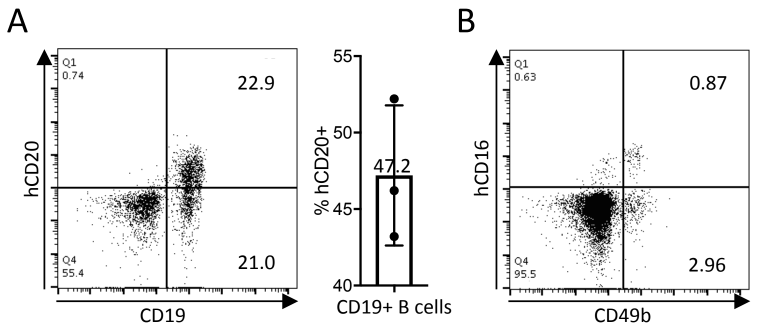

Supplementary Figure 3
Graphical Abstract

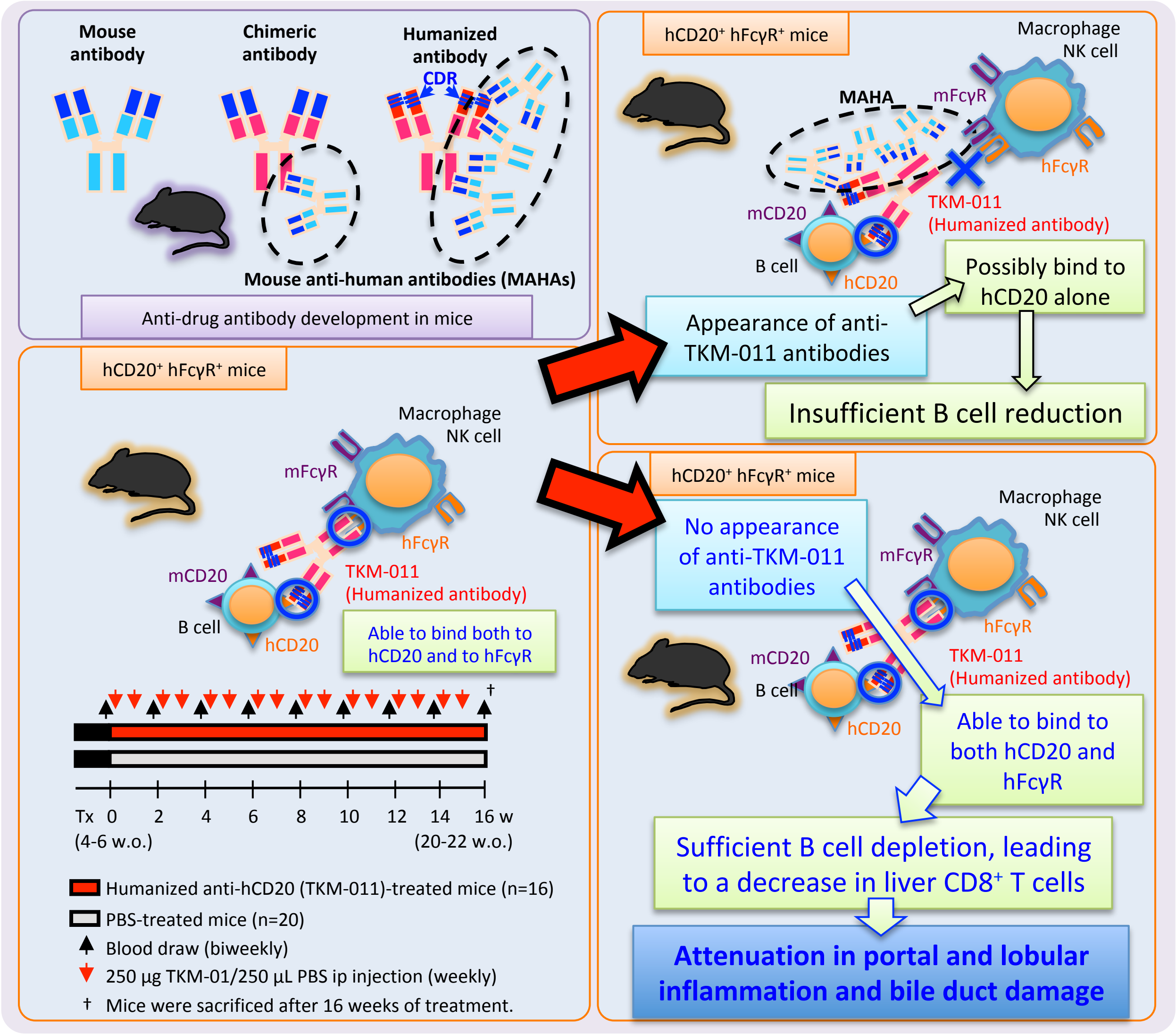

Supplementary Figure 4

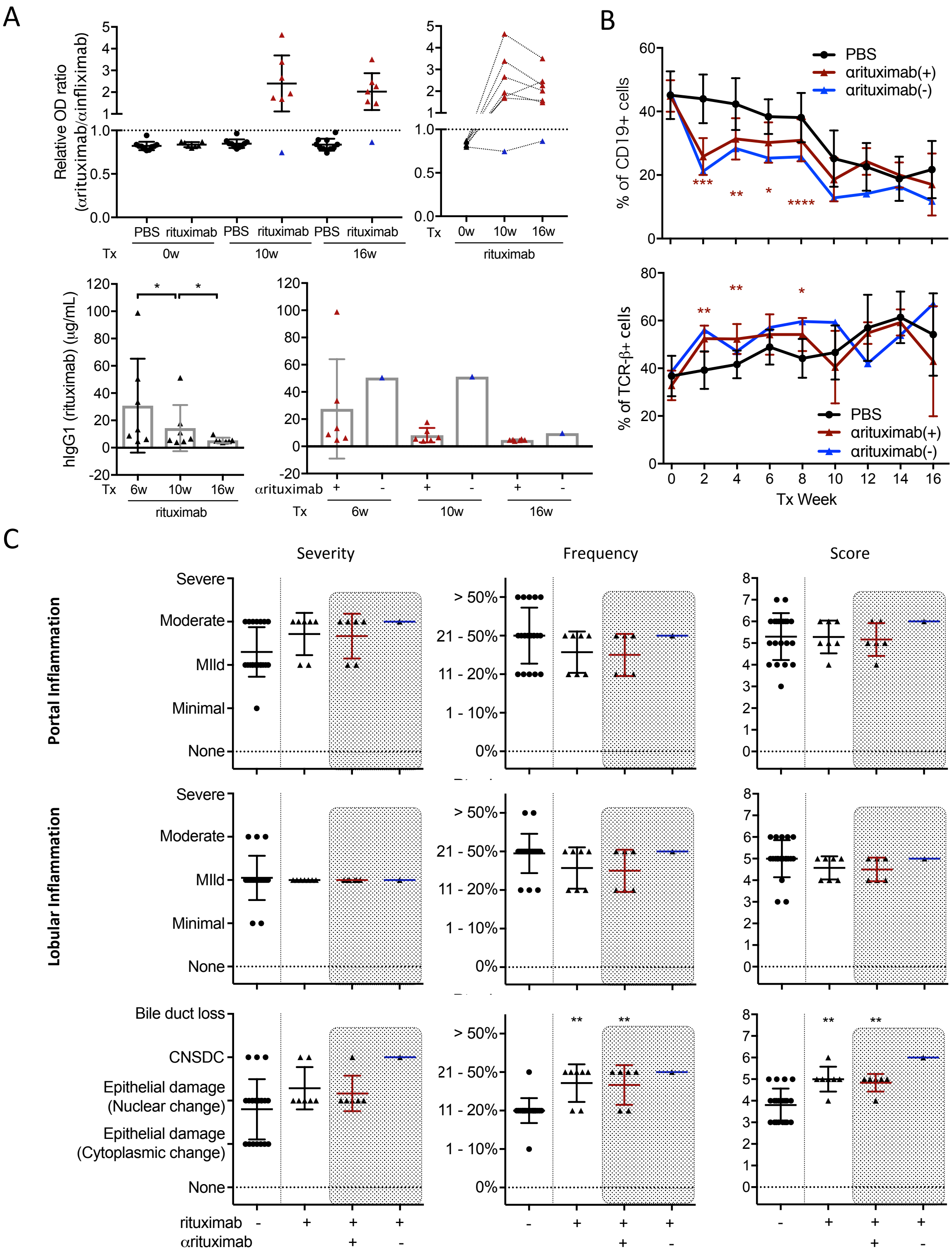

Supplement: Supplementary Figure 1 — Splenic B-cell depletion in human CD20 expressing BALB/c mice in the presence or absence of human FcγR expression. (A,B) Representative flow cytometry dot plots. Splenic mononuclear cells (MNCs) were pre-incubated with mouse FcR blocking reagent and then incubated at 4°C with a combination of fluorochrome-conjugated antibodies (BD Biosciences), including FITC-conjugated anti-CD45R/B220, and PE-conjugated anti-human CD20 (hCD20) as well as FITC-conjugated anti-mouse CD49b/DX5 and PE-conjugated anti-human CD16 (hCD16, hFcγRIII). Cell-surface expression of hCD20 in CD45R+ B cells was observed in hCD20-expressing mice regardless of hFcγR expression. Cell-surface expression of hCD16 in CD49b+ NK cells was observed in hCD20- and hFcγR-expressing mice, but not in hFcγR-negative mice. (C) Anti-hCD20 humanized antibody TKM-011 (250 μg in 250 μL of PBS) and the chimeric antibody rituximab (250 μg in 250 μL of PBS) or 250 μL of PBS alone (as a control) were injected intraperitoneally into hCD20-expressing BALB/c mice in the presence or absence of hFcγR expression. Spleens were extracted 7 days after the injection. Splenic MNCs were counted, and an aliquot of these cells was stained as shown above and analyzed using flow cytometry. Absolute numbers of total CD19+ cells were calculated. Enhanced B-cell depletion was observed in mice expressing both hCD20 and hFcγR, suggesting an in vivo functional mechanism of hFcγR in mediating antibody-dependent cell-mediated cytotoxicity (ADCC). [file Data_Sheet_1.pdf]
